# Light chain deposition disease presenting as paroxysmal atrial fibrillation: a case report

**DOI:** 10.1186/1752-1947-1-187

**Published:** 2007-12-29

**Authors:** Fabio Fabbian, Nevio Stabellini, Sergio Sartori, Paola Tombesi, Arrigo Aleotti, Maurizio Bergami, Simona Uggeri, Adriana Galdi, Christian Molino, Luigi Catizone

**Affiliations:** 1 Renal Unit, St. Anna Hospital, Ferrara, Italy; 2 Internal Medicine, University of Ferrara, Ferrara, Italy; 3 Electron Microscopy Service§ University of Ferrara, Ferrara, Italy

## Abstract

**Introduction:**

Light chain deposition disease (LCDD) can involve the heart and cause severe heart failure. Cardiac involvement is usually described in the advanced stages of the disease. We report the case of a woman in whom restrictive cardiomyopathy due to LCDD presented with paroxysmal atrial fibrillation.

**Case presentation:**

A 55-year-old woman was admitted to our emergency department because of palpitations. In a recent blood test, serum creatinine was 1.4 mg/dl. She was found to have high blood pressure, left ventricular hypertrophy and paroxysmal atrial fibrillation. An ACE-inhibitor was prescribed but her renal function rapidly worsened and she was admitted to our nephrology unit. On admission serum creatinine was 9.4 mg/dl, potassium 6.8 mmol/l, haemoglobin 7.7 g/dl, N-terminal pro-brain natriuretic peptide 29894 pg/ml. A central venous catheter was inserted and haemodialysis was started. She underwent a renal biopsy which showed kappa LCDD. Bone marrow aspiration and bone biopsy demonstrated kappa light chain multiple myeloma. Echocardiographic findings were consistent with restrictive cardiomyopathy. Thalidomide and dexamethasone were prescribed, and a peritoneal catheter was inserted. Peritoneal dialysis has now been performed for 15 months without complications.

**Discussion:**

Despite the predominant tubular deposition of kappa light chain, in our patient the first clinical manifestation of LCDD was cardiac disease manifesting as atrial fibrillation and the correct diagnosis was delayed. The clinical management initially addressed the cardiovascular symptoms without paying sufficient attention to the pre-existing slight increase in our patient's serum creatinine. However cardiac involvement is a quite uncommon presentation of LCDD, and this unusual case suggests that the onset of acute arrhythmias associated with restrictive cardiomyopathy and impaired renal function might be related to LCDD.

## Introduction

Light chain deposition disease (LCCD) is a systemic disease involving several organs. Kidney impairment usually dominates the clinical picture, and proteinuria and renal failure are the most common clinical manifestations [1]. Heart involvement plays a crucial role in the prognosis of the disease; signs and symptoms of cardiac dysfunction are related to the degree of myocardial deposition of light chains, and generally occur in the advanced stages of the disease. We report a case of LCCD in which paroxysmal atrial fibrillation was the first clinical manifestation.

## Case presentation

In December 2006, a 55-year-old woman was admitted to the emergency department of our hospital because of palpitations. She had a history of cholecystectomy because of biliary stone, hysterectomy and hypothyroidism treated with thyroxine 50 μg/day. In a laboratory test performed in October 2006, serum creatinine was 1.4 mg/dl and urine analysis was normal. Clinical examination showed hypertension and atrial fibrillation. Echocardiography demonstrated left ventricular hypertrophy and diastolic dysfunction, with normal ejection fraction. The atrial fibrillation resolved spontaneously, and treatment with an angiotensin converting enzyme (ACE)-inhibitor was started. In January 2007, a further episode of atrial fibrillation occurred. It resolved after intravenous propafenone, but serum laboratory tests showed an increase in creatinine (3 mg/dl) and potassium levels (5.8 mmol/l), and the ACE-inhibitor was stopped. Thyroid function was normal. Three weeks later, serum creatinine was found to have further increased up to 5 mg/dl, whereas both kidneys appeared normal on sonography (US) examination. The patient was admitted to our nephrology unit. On admission serum creatinine was 9.4 mg/dl (normal reference values 0.7–1.3), potassium 6.8 mmol/l (normal reference values 3.7–5.3), and haemoglobin 7.7 g/dl (normal reference values 11.5–16.5). Two units of packed red cells were transfused, a central venous catheter was inserted, and haemodialysis was started. Proteinuria was 1 g/day and urine sediment analysis showed haematuria. Serum glucose was 85 mg/dl (normal reference values 70–110), sodium 140 mmol/l (normal reference values 136–146), calcium 2.4 mmol/l (normal reference values 2.15–2.55), proteins 7.1 g/dl (normal reference values 6.6–8.7), and albumin 43 g/L (normal reference values 35–46); protein electrophoresis did not show any monoclonal spike, IgG was 630 mg/dl (normal reference values 600–1600), IgA 71 mg/dl (normal reference values 70–400), IgM 44 mg/dl (normal reference values 40–230), C3 132 mg/dl (normal reference values 90–180), C4 54 mg/dl (normal reference values 16–38), autoantibodies were negative and N-terminal pro-brain natriuretic peptide (NT-proBNP) was 29894 pg/ml (Roche, Indianapolis, IN, USA; normal reference values <247 pg/ml). Immunofixation showed monoclonal kappa light chain in the urine. Echocardiography detected substantial thickening of the left wall in the septum and posterior wall and diastolic ventricular dysfunction, findings suggestive of restrictive cardiomyopathy.

**Figure 1:**
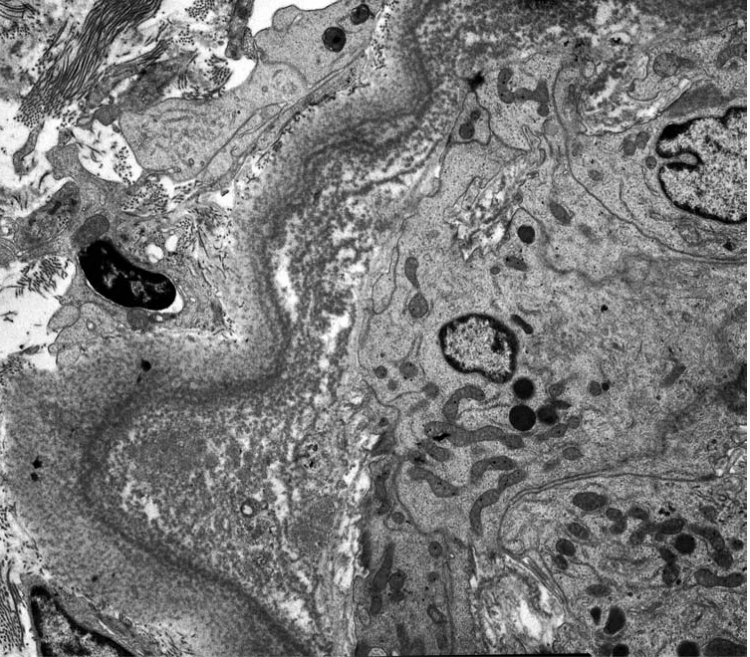
Electron microscopy photograph showing coarsely granular electron-dense deposits along the outer surface of the tubular basement membrane.

**Figure 2:**
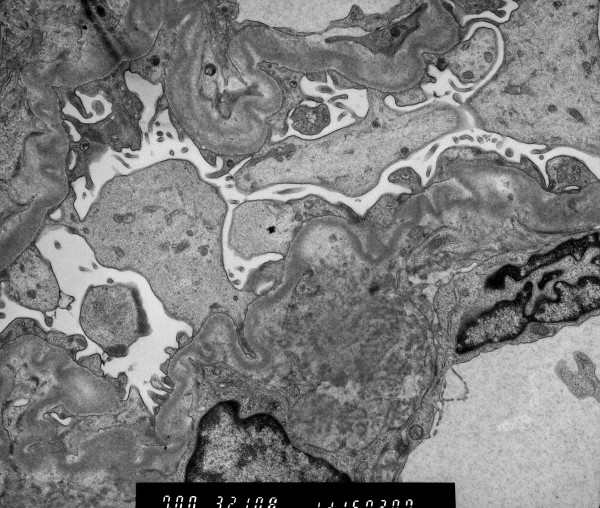
Electron microscopy photograph showing nonfibrillar electron-dense deposits in the endothelial side of the glomerular basement membrane.

The patient underwent US-guided biopsy of the lower pole of the right kidney, and two specimens were obtained for light and electron microscopy examination. Light microscopy examination showed smooth and continuous deposition of eosinophil material in the tubular basement membrane, mild thickening and stiffness of the glomerular basement membrane, and increase of the mesangial matrix. Congo red stain was negative, but immunofluorescence revealed linear deposits of kappa light chains within the tubular basement membranes. Electron microscopy examination displayed coarse granular electron-dense deposits in the outer surface of the tubular basement membranes (Figure 1), and nonfibrillar electron dense material along the glomerular basement membrane and in the mesangium (Figure 2). Bone marrow aspiration and bone biopsy were performed, and histologic examination of the specimens confirmed the diagnosis of monoclonal immunoglobulin deposition disease associated to kappa light chain multiple myeloma. Treatment with thalidomide 100 mg/day and dexamethasone 40 mg on days 1–4 every 28 days was started, a peritoneal catheter was inserted, and the patient was changed from haemodialysis to peritoneal dialysis. At the time of writing the patient has been dialysing for 15 months and no major complications have been recorded.

The study has been conducted according to the Declaration of Helsinky.

## Discussion

The pathogenesis of LCDD is similar to that of primary amyloidosis. Both are monoclonal plasma cell proliferative disorders characterized by tissue deposition of light chain fragments, leading to organ impairment. Myeloma is diagnosed in about 40% of patients with LCDD [2]. In LCDD light chain fragments do not form amyloid fibrils; they are defined as non-amyloid immunoglobulin light chains, and are mostly kappa chains. They cause renal failure and extra-renal manifestations usually secondary to heart, liver and peripheral nerve involvement [3]. Survival is quite variable, ranging from 1 month to 10 years from the onset of symptoms, and mortality is mainly due to heart or liver failure, or progression to multiple myeloma [2]. Serum creatinine at the time of diagnostic renal biopsy seems to be the only predictive factor of renal function and patient survival [1,3].

In plasma cell disorders with dysproteinemia, the aggregation of non-amyloid immunoglobulin light chains forms granular deposits, diffusely distributed in systemic basement membranes, which suggest a mechanism of aggregation and deposition different from primary amyloidosis [4]. Several cofactors have been suggested to play a role in promoting fibrilogenesis, such as the binding to subunit proteins, the stabilization of fibrils, and their protection from degradation [5]. In the kidneys, LCDD is often associated with deposits in the tubular basement membranes and Bowman's capsule, which may be more prominent than those deposits seen in the glomeruli. Clinical presentation depends in part on the site of deposition: it follows that patients with predominant glomerular deposition develop nephrotic syndrome, while those with predominant tubular deposition develop renal failure and mild proteinuria [2]. In most cases, renal function declines rapidly, with the clinical features of subacute tubulointerstitial nephritis or rapidly progressive glomerulonephritis [3]. In our patient, the clinical presentation showed similar characteristics, but it was initially misunderstood. An ACE-inhibitor was prescribed without paying sufficient attention to the slight increase in creatinine levels, and the subsequent worsening of renal function was ascribed to that medication. Such a misunderstanding delayed the correct diagnosis, and the clinical management was erroneously focussed on the cardiac manifestations. Indeed, atrial fibrillation associated with restrictive cardiomyopathy is a quite uncommon presentation of LCDD, and the patient was referred to the nephrology unit only when the renal disease impairment became severe.

Endomyocardial biopsy is the gold standard to demonstrate heart involvement in LCDD. Histologic examination of deep-frozen specimens shows amorphous Congo red-negative deposits that are positive for light chains on immunofluorescence [6,7]. Clinical manifestations of heart involvement are variable and similar to those observed in restrictive cardiomyopathy induced by amyloidosis. They can range from congestive heart failure to arrhythmias and conduction disorders; myocardial infarction has also been reported [8,9]. Recently Toor et al. [10] described cardiac nonamyloidotic immunoglobulin deposition disease in 8 patients who underwent endomyocardial biopsy. The median age was 49.5 years, none of them were symptomatic and on echocardiography six patients had concentric left ventricular hypertrophy with diastolic dysfunction. One of them developed atrial fibrillation during chemotherapy and responded to therapy with digoxin.

In our patient left ventricular morphology and the transmitral inflow pattern demonstrated by doppler echocardiography were consistent with diastolic ventricular dysfunction due to restrictive cardiomyopathy [11]. Response to treatment may differ between amyloidosis-induced and LCDD-induced cardiomyopathy, and the latter could resolve after successful treatment of the underlying plasma cell disorder [7]. However, whatever signs and symptoms may reveal heart impairment in LCDD, they usually occur in the advanced stages of the disease.

To our knowledge, atrial fibrillation associated with restrictive cardiomyopathy has never been previously reported in the medical literature as a first clinical manifestation of restrictive cardiomyopathy due to LCDD. Although Palladini et al. [12] demonstrated that NT-proBNP assay can be useful in detecting cardiac involvement in amyloidosis, we observed that natriuretic peptide levels were no more effective than echocardiography in evaluating heart disease in a patient with primary amyloidosis and uraemia [13]. However, the very high levels of NT-proBNP observed in the patient in this present case report could have been the result of the combination of heart involvement and impaired renal function, as renal failure can influence NT-proBNP assay performed by Roche method [14].

In conclusion, this unusual case suggests that the onset of acute arrhythmias associated with restrictive cardiomyopathy and impaired renal function might be related to LCDD.

## Competing interests

The author(s) declare that they have no competing interests.

## Authors' contributions

FF, NS, MB, SU, AG, CM, LC performed the clinical work and made the diagnosis, acquired, analyzed and interpreted the data.

FF and SS drafted the manuscript.

SS, PT performed the investigations.

AA performed the electron microscopy work.

Every author reviewed and approved the manuscript which is not under consideration for publication elsewhere in a similar form, in any language.

## Consent

The patient was informed about the article and hypothetical beneficial effects of its publication on clinical practice. Written informed consent was obtained from the patient for publication of this Case report and any accompanying images. A copy of the written consent is available for review by the Editor-in-Chief of this journal.
